# New Beta-lactamases in Candidate Phyla Radiation: Owning Pleiotropic Enzymes Is a Smart Paradigm for Microorganisms with a Reduced Genome

**DOI:** 10.3390/ijms23105446

**Published:** 2022-05-13

**Authors:** Mohamad Maatouk, Ahmad Ibrahim, Lucile Pinault, Nicholas Armstrong, Said Azza, Jean-Marc Rolain, Fadi Bittar, Didier Raoult

**Affiliations:** 1 IHU Méditerranée Infection, 13005 Marseille, France; mohamad.maatouk@etu.univ-amu.fr (M.M.); ahmad.ibrahim@etu.univ-amu.fr (A.I.); lucile.pinault@gmail.com (L.P.); nicholas.armstrong@univ-amu.fr (N.A.); said.azza@univ-amu.fr (S.A.); jean-marc.rolain@univ-amu.fr (J.-M.R.); fadi.bittar@univ-amu.fr (F.B.); 2 Aix-Marseille Université, IRD, APHM, MEPHI, 13005 Marseille, France

**Keywords:** antibiotic resistance, beta-lactamase, candidate phyla radiation, multifunction hydrolase enzymes, RNase

## Abstract

The increased exploitation of microbial sequencing methods has shed light on the high diversity of new microorganisms named Candidate Phyla Radiation (CPR). CPR are mainly detected via 16S rRNA/metabarcoding analyses or metagenomics and are found to be abundant in all environments and present in different human microbiomes. These microbes, characterized by their symbiotic/epiparasitic lifestyle with bacteria, are directly exposed to competition with other microorganisms sharing the same ecological niche. Recently, a rich repertoire of enzymes with antibiotic resistance activity has been found in CPR genomes by using an in silico adapted screening strategy. This reservoir has shown a high prevalence of putative beta-lactamase-encoding genes. We expressed and purified five putative beta-lactamase sequences having the essential domains and functional motifs from class A and class B beta-lactamase. Their enzymatic activities were tested against various beta-lactam substrates using liquid chromatography-mass spectrometry (LC-MS) and showed some beta-lactamase activity even in the presence of a beta-lactamase inhibitor. In addition, ribonuclease activity was demonstrated against RNA that was not inhibited by sulbactam and EDTA. None of these proteins could degrade single- and double-stranded-DNA. This study is the first to express and test putative CPR beta-lactamase protein sequences in vitro. Our findings highlight that the reduced genomes of CPR members harbor sequences encoding for beta-lactamases known to be multifunction hydrolase enzymes.

## 1. Introduction

Advances in sequencing methods have contributed to the exploration of microbial dark matter [1,2]. A new microbial division, close to bacteria, has recently been described and termed Candidate Phyla Radiation (CPR) [3,4]. Members belonging to the CPR division represent more than 26% of known microbial diversity [2]. They have a small size (100 to 300 nm), a reduced genome (around one megabase pair) [5], as well as a high percentage of hypothetical proteins without known biosynthetic and metabolic activities [6]. Moreover, they are characterized by their unique lifestyle of obligatory exo-symbiotic/parasitic relationships with bacteria [6,7,8]. To date, few studies have succeeded in co-culturing some CPR species with their bacterial hosts [9,10,11,12,13]. One co-culture was obtained by adding kanamycin to the enrichment broth for a TM7 strain [9]. Moreover, another study showed that a co-culture was also achieved with streptomycin selection of CPR and its bacterial host [10].

Through metabarcoding analysis of 16S rRNA and whole metagenomics, CPR have been detected in different environments and ecological niches around the world and have more than 73 phyla [14,15,16,17,18,19]. Until now, only three CPR superphyla have been found among the human microbiome: Saccharibacteria, Gracilibacteria, and Absconditabacteria superphyla [14,20,21,22,23,24,25,26].

Microbial diversity creates competition for nutrients and space in the different environments they inhabit [27]. When sharing the same habitat with others, the effectiveness of antimicrobial agents secreted by microorganisms enables their growth and improves their chances of survival [28]. The secretion of these compounds and the carrying of antibiotic resistance genes are ancient phenomena [29,30]. These phenomena allow for the rapid accumulation of numerous resistance variations at a relatively high evolutionary rate [31]. Although CPR have been around for a very long time, their detection has recently been revealed by metagenomic analysis. They have been detected in ancient samples of dental calculus [32,33,34].

A recent study has shown through extensive in silico analysis that all tested CPR superphyla were well equipped with highly diverse enzymes with putative antibiotic resistance activities [35]. Following the application of relaxed parameters on CPR protein sequences, they finally regrouped 30,545 encoding enzymes with functional domains that linked to 14 different chemical classes of antimicrobials. The CPR resistome involved mostly beta-lactam, glycopeptide, and macrolide–lincosamide–streptogramin resistance families (MLS) [35]. Thus, carrying multifunctional coding genes could be an advantage in microorganisms with a small-size genome [36].

Beta-lactamases are very diverse multifunctional enzymes [37]. Beta-lactam antibiotics function mainly by blocking the synthesis of peptidoglycan in bacteria [38]. To date, more than 4000 bacterial beta-lactamases have been described [39] and classified into four classes: class A, C, D (called serine beta-lactamase), and class B (called metallo-beta lactamase). These enzymes have different functions such as beta-lactam antibiotic inactivation, arylsulfatase, glyoxalase II, alkyl sulfatase, cyclase, CMP-NeuAc hydroxylase, cAMP phosphodiesterase, flavoproteins, phosphonate metabolism proteins, cleavage and polyadenylation specific factors, DNA cross-link repair proteins, ribonuclease, and others [40]. They are pleiotropic proteins with both native and promiscuous activities. Moreover, the genome annotation of different life domains showed the presence of protein-encoding genes with beta-lactamase activity in archaea, eukaryotes (in the human genome), some giant viruses, and bacteriophages [36,40,41,42,43]. Therefore, the objective of this research was to explore in vitro the multifunctional hydrolase activities of selected putative beta-lactamase-encoding genes detected in the CPR resistome against different antibiotic agents, DNA and RNA substrates.

## 2. Results

### 2.1. CPR Putative Beta-Lactamase-Encoding Genes

We selected five of the most repetitive CPR putative beta-lactamase-encoding genes from the CPR resistome (see Materials and Methods section) [35]. These sequences have the necessary functional domain for beta-lactamase activity. All domains were detected according to CDD (conserved domains database) search with maximum e-value 0.0001. The manual research for active sites/motifs allowed us to detect in each sequence of class A beta-lactamase S-x-x-K, S-D-N, and K-T-G, and for class B beta-lactamase H-x-H-x-D-H (where x can be any amino acid) [44]. The existence of beta-lactamase domains and motifs indicates that A-1, A-2, B-1, B-2, and B-3 are probably functional enzymes with beta-lactamase activity. Furthermore, these selected sequences showed high confidence (100%) for homology/analogy with other proteins recognizing hydrolase beta-lactamase activity, according to the 3D structure prediction using the Phyre^2^ investigator database. All sequences were similar in size to the known bacterial beta-lactamase except B-3, which was three times larger than average. However, these sequences showed a low similarity with the bacterial origin of beta-lactamase (average of 27.6% [23.1−36.6%]). Further information about each selected CPR sequence is listed in Table 1 (see also Table S1 in the supplemental material).

### 2.2. Antibiotic Susceptibility Testing

We performed the E-test on the five *Escherichia coli* carrying the CPR sequence coding the putative beta-lactamase protein using different beta-lactam antibiotics in triplicate (see Materials and Methods section). Compared to the *E. coli* sensitive strain (BL21(DE3)-pGro7/GroEL), we did not notice any change in the inhibition zone or the minimum inhibitory concentration (MIC) values. The MIC value was 0.75 µg/mL for ampicillin, 1.5 µg/mL for amoxicillin, 8 µg/mL for benzylpenicillin, less than 0.016 µg/mL for cefepime, less than 0.016 µg/mL for ceftriaxone, 0.023 µg/mL for ceftazidime, 0.064 µg/mL for ceftolozane, 0.19 µg/mL for imipenem, less than 0.125 µg/mL for meropenem, 0.003 µg/mL for ertapenem, and less than 0.016 µg/mL for aztreonam. All *E. coli* remained sensitive to all tested beta-lactam antibiotics.

### 2.3. β-. Lactam-Hydrolyzing Activity

**Figure 1:**
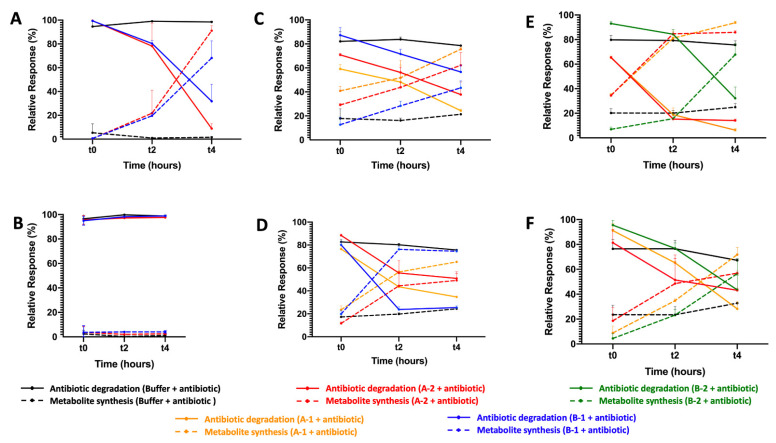
Enzymatic test of CPR putative beta-lactamases measured by LC-MS. (**A**) Beta-lactamase activity test against penicillin G in the absence of clavulanic acid, (**B**) and in the presence of clavulanic acid. (**C**) Beta-lactamase activity test against amoxicillin in the absence of clavulanic acid, (**D**) and in the presence of clavulanic acid. (**E**) Beta-lactamase activity test against ampicillin in the absence of clavulanic acid (**F**) and in the presence of clavulanic acid. Only CPR putative beta-lactamases that degrade beta-lactam substrates were shown.

In addition, after expressing the putative CPR beta-lactamase-encoding genes in *E. coli* strains and obtaining an adequate volume of pure proteins, the activity of each one (A-1, A-2, B-1, B-2, and B-3) was tested against several beta-lactam substrates. This activity was monitored using the liquid chromatography-mass spectrometry (LC-MS) in the presence and absence of clavulanic acid as a beta-lactam inhibitor. Regarding penicillin beta-lactams, when testing the hydrolase activity of the five CPR proteins against penicillin G, only B-1 and A-2 were found to have penicillinase activity, with a significant decrease in penicillin content level (Figure 1). A-2 had stronger activity than B-1 against penicillin G, with a greater and faster formation of its metabolite benzylpenilloic acid. However, after adding clavulanic acid, both enzymes A-2 and B-1 were inhibited when the level of penicillin G remained stable throughout the experiment. In contrast, the addition of clavulanic acid did not totally inhibit the hydrolase activity of the CPR enzymes, which were able to degrade amoxicillin in its absence. A-1, A-2, and B-1 had a significant capacity to degrade amoxicillin and to promote the synthesis of its metabolite, the amoxilloic acid, unlike B-2, which did not have an effect (Figure 1). Even though clavulanic acid did not prevent degradation of amoxicillin, it seems like it can accelerate the hydrolase activity of B-1 and slightly that of A-2.

Moreover, A-1, A-2, and B-2 significantly hydrolyzed ampicillin and formed ampicilloic acid even in presence of beta-lactamase inhibitor clavulanic acid (Figure 1). Clavulanic acid did slightly slow down the hydrolase of ampicillin by A-2 and B-2, with less degraded ampicillin during the experiment. For the CPR putative protein B-1, there was no effect on ampicillin. Note that B-3 had no hydrolase activity against the beta-lactam antibiotics penicillin G, ampicillin, and amoxicillin. For the other beta-lactam antibiotic tests, the five CPR putative beta-lactamases had no activity against cefotaxime and imipenem, representative of cephalosporin and carbapenem beta-lactam classes (data not shown).

### 2.4. Characterization of the DNase and RNase Activities

**Figure 2:**
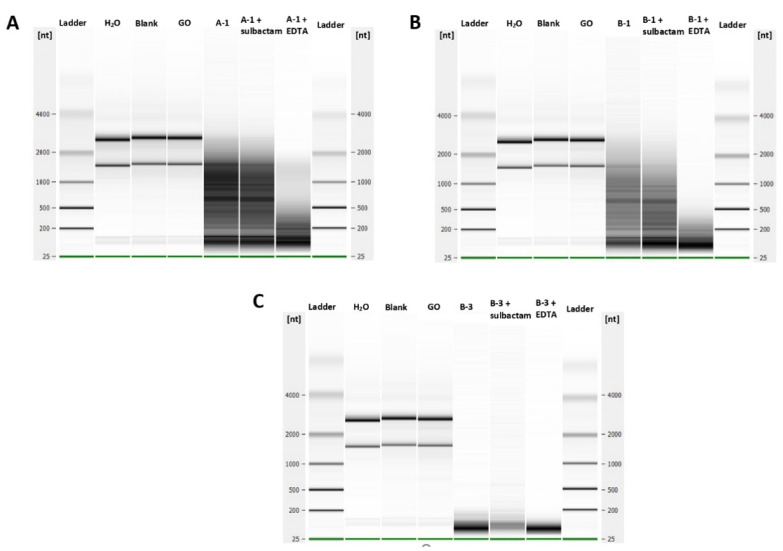
Ribonuclease activities of the purified CPR tested proteins. (**A**) Degradation of bacterial total RNAs by the A-1 CPR enzyme, (**B**) B-1 CPR enzyme, and (**C**) B-3 CPR enzyme in the presence and absence of a metallo-beta-lactamase inhibitor (sulbactam and EDTA). GO (glycine oxidase) was used as a negative control enzyme.

Finally, the purified CPR proteins were used to assess their DNase and RNase activities in vitro. We found no nuclease activity on single- or double-stranded DNA for all CPR proteins tested (A-1, A-2, B-1, B-2, and B-3) (data not shown) compared to its ribonuclease activities. In fact, after 2 h of incubation, the RNA of *E. coli* was partially hydrolyzed with the putative beta-lactamases A-1 and B-1 and completely hydrolyzed with the putative beta-lactamase B-3 (Figure 2). This RNase activity was not inhibited by the beta-lactamase inhibitor sulbactam or the EDTA chelator (Figure 2). The use of glycine oxidase (GO) as a negative enzyme control with the same conditions of expression and purification used for CPR proteins had no RNase activity, as expected.

## 3. Discussion

CPR members are newly described microbes with few features/facts known concerning their genomic contents and biosynthetic capabilities. Here, we focused on the “hidden” putative beta-lactamases detected in CPR genomes [35], currently annotated as an unknown function. In this work, we showed that putative beta-lactamase-encoding genes detected in the CPR resistome have multifunctional hydrolase activities against some beta-lactam antibiotics and RNA in vitro.

Beta-lactamase enzyme genes have been reported in bacteria, archaea, giant viruses, and some eukaryotes, including the human genome [36,40,42,43]. However, putative beta-lactamase-encoding genes in CPR genomes have recently been reported in the literature [35]. Their activities on beta-lactam antibiotics have not been investigated. For CPR, we suggest that detected putative beta-lactamase-encoding genes are not only used for beta-lactam hydrolysis/degradation. They are also used for other activities, such as RNase for example, exactly like those detected in human DNA [40]. This may explain the abundance of putative beta-lactamases in CPR cells, since their genome is reduced (with a limited number of encoding genes) and may need multifunctional encoding genes to perform various activities. Moreover, the activity of clavulanic acid was not the same on the CPR tested enzymes against the different beta-lactam substrates. It did not prevent the degradation of amoxicillin and ampicillin as the case of penicillin G, but it did influence the hydrolysis reaction of the beta-lactam substrates. It may have had an accelerating effect in the case of amoxicillin for two of the enzymes A-2 and B-1 and a slowing down effect in the case of ampicillin for A-2 and B-2. However, our hypothesis on these enzymes and its interaction with other beta-lactamase inhibitors needs to be explored, since the significance of beta-lactamase enzyme genes in CPR microbes is not yet known.

Additionally, RNase activity was detected in CPR beta-lactamase sequences A-1, B-1, and B-3. Thus, B-3 has three times the length of average bacterial beta-lactamases with different motifs (data not shown). This ribonuclease activity has been described with multi-motifs and can be used for the formation of different proteins with several functions, depending on the splicing site of their RNAs having intron sequences, such as the case of some CPR genomes [3,15,45].

Moreover, the putative beta-lactamase-encoding genes detected in CPR can play a protective role for its bacterial host against some antibiotics. This may explain why most bacteria found in the oral cavity (where CPR is more abundant) are phenotypically sensitive to beta-lactam antibiotics [46]. This protective role has also been reported phenotypically after the successful co-culture of CPR in the presence of aminoglycoside antibiotics [9,47]. Indeed, there is currently no phenotypic test to detect the sensitivity or resistance of CPR to beta-lactam substrates. Only the realization of such a test in association with their symbiont will allow identification of the role of beta-lactamase for the direct protection of CPR or its host.

Our findings suggest that the sensitivity of the *E. coli* strain carrying the putative CPR beta-lactamase-encoding gene to different beta-lactam antibiotics could be due to its low expression through bacteria. The obtained concentration of all proteins after purification was low compared to other proteins purified following the same bacterial quantity/protocol, which confirms the poor expression of our beta-lactamase genes by *E. coli* [40].

## 4. Materials and Methods

### 4.1. Choice of Expressed Sequences

A total of 5759 amino acid sequences with putative beta-lactamase activity (Class A: 1606; B: 3359; C: 27, and D: 767) were retrieved from the CPR resistome according to Maatouk et al., [35]. For in vitro verification, we selected from the most abundant beta-lactamase classes the most repetitive sequences: 2 protein sequences of class A putative beta-lactamases (A-1 and A-2, repeated 16 times each) and 3 protein sequences of class B putative beta-lactamases (B-1 repeated 7 times, B-2 repeated 13 times, and B-3 repeated 4 times each). In addition, before expression, we researched the presence of motifs in each sequence, using a motif search online tool [48]. Then, the 3D structure of the selected CPR putative beta-lactamase was generated and analyzed to predict its function in silico using the Phyre^2^ tool [49,50]. A1, A2, B-2 and B-3 were annotated as hypothetical proteins based on the RAST server annotation tool [51]. However, B1 was annotated as a metallo-beta-lactamase-fold hydrolase superfamily.

### 4.2. Sequence Cloning, Expression, and Protein Purification

Each nucleic acid of the five selected CPR sequences was optimized to the GC% of the E. *coli* genome for bacterial expression in vitro. All modified sequences were synthesized by GenScript (https://www.genscript.com/, accessed on 10 March 2021) (Piscataway, NJ, USA) and cloned into the pET24a( + ) expression plasmid, between NdeI and NotI restriction sites. In addition, a Strep-tag was added at the N-terminal of each cloned sequence. We made this step in order to facilitate the selection of the protein of interest and to ensure its purity. One μL of plasmidic DNA was electroporated into electrocompetent *E. coli* BL21(DE3)-pGro7/GroEL (Takara, Kyoto, Japan) and recovered by adding 1 mL Luria-Bertani (LB) broth (Becton, Dickinson and Company, Le Pont de Claix, France), followed by incubation at 37 °C for 1 h. Then, 100 µL were plated on LB agar (Becton, Dickinson and Company, Le Pont de Claix, France) supplemented by 12 µL (50 mg/mL) kanamycin (Gibko, Loughborough, United Kingdom) for selection. Selected colonies were inoculated in LB media supplemented by kanamycin (50 mg/mL), exactly as previously described [52].

Grown clones were enriched in ZYP-5052 auto-induction medium at 37 °C with agitation (180 rpm), until it reached an O.D._600 nm_ = 0.6, which represents the exponential growth phase of *Escherichia coli* [53]; thereafter, the incubation temperature was reduced to 20 °C for an additional 20 h. Then, centrifugation (5000× g) was performed for 30 min at 8 °C and each pellet was resuspended in 50 mL washing buffer (50 mM Tris pH 8, 300 mM NaCl) and stored overnight at −80 °C. Then, we added 0.25 mg/mL lysozyme, 10 µg/mL DNase I, and 0.1 mM PMSF (phenylmethylsulfonyl fluoride) to each frozen *E. coli* and incubated on ice for 1 h. The partially lysed bacteria were disrupted by three sonication cycles (30 s, amplitude 45) performed on a Q700 sonication system (QSonica, Newtown, Connecticut, USA). Centrifugation was then carried out to eliminate all bacterial debris (10,000 g, 20 min, 8 °C). Protein purification was performed using the affinity Strep-tag chromatography (wash buffer: 50 mM Tris pH 8, 300 mM NaCl, and elution buffer: 50 mM Tris pH 8, 300 mM NaCl, 2.5 mM desthiobiotin) on a 5 mL StrepTrap HP column (GE Healthcare). Fractions containing each protein of interest were pooled. Protein purity was assessed using 12.5% SDS-PAGE analysis (Coomassie staining). Finally, each recombinant protein concentration was measured using a Nanodrop 2000c spectrophotometer (Thermo Scientific, Madison, WI, USA).

### 4.3. Antibiotic Susceptibility Testing

For beta-lactam antibiotic susceptibility testing, each *E. coli* with OD = 0.6, 0.01 g/mL of IPTG (isopropylthiogalactoside) (Euromedex, Strasbourg, France) was added to 1 mL of the broth to induce protein expression. Then, a 0.5 McFarland solution was prepared for each one and cultured on MH (Muller Hinton) agar (BioMérieux, Marcy l’Étoile, France). The MIC of ampicillin, amoxicillin, benzylpenicillin, cefepime, ceftriaxone, ceftazidime, ceftolozane, imipenem, meropenem, ertapenem, and aztreonam was determined by the E-test method (BioMérieux, Marcy l’Étoile, France), as per the recommendation of the European Committee on Antimicrobial Susceptibility Testing (https://www.eucast.org). Results were visualized after 24 h of incubation. Uninoculated and non-transformed *E*. *coli* BL-21 culture and control cultures without adding beta-lactam antibiotics were used as negative controls.

### 4.4. Liquid Chromatography-Mass Spectrometry: Beta-Lactam Antibiotic Degradation Assay

To determine the beta-lactam hydrolysis activity of the selected CPR proteins, a stock solution of the following antibiotics was freshly prepared in water from the respective high-purity salts: penicillin G, amoxicillin, ampicillin, cefotaxime, and imipenem (Sigma Aldrich, Saint-Louis, MO, USA). Each purified protein solution was spiked with 1 μL of each antibiotic mentioned above with/without clavulanic acid at a final concentration of 10 μg/mL (mass concentration equivalent for the evaluated antibiotic) and all mixtures were incubated at room temperature. To stop the reaction, we added acetonitrile to each sample (70% of the final volume). Then, a 5 min centrifugation step at 12,100× *g* was performed for protein precipitation. The supernatant was collected for analysis. This assay was prepared in triplicate for each sample and measured at 3 different incubation times (t = 0, t = 2 h, and t = 4 h). We used PBS (phosphate-buffered saline) mixed with each tested antibiotic as a negative control. However, we could not use any positive control since it is the first in vitro characterization of CPR enzymes, and their sequences are not comparable with those in the literature.

Then, for the data interpretation, the MS responses of each substrate was normalized using the MS response of cilastatine (ratio). The analysis was conducted using an ACQUITY I-Class UPLC chromatography system connected to a Vion IMS QTOF ion mobility-quadrupole-time of flight mass spectrometer as previously described [36]. The significant hydrolysis activity of the CPR tested proteins was measured using the Wilcoxon signed ranks statistic test method.

### 4.5. DNase and RNase Activity Evaluation

The DNase activity of the putative beta-lactamase enzymes was evaluated using synthesized single-stranded reverse and forward DNAs and double-stranded DNAs of 130-pb as substrates. Double-stranded DNA was generated by annealing forward and reverse single-stranded DNA in a thermocycler by decreasing temperatures from 95 to 25 °C for 1 h. The enzymatic reactions were performed by incubating each polynucleotide (1 μg each) with 15 μg of the expressed putative beta-lactamase protein in 1 × CutSmart Buffer (New England Biolabs, Massachusetts, USA), 50 mM potassium acetate, 20 mM tris–acetate, 10 mM magnesium acetate, and 100 g/mL BSA (bovine serum albumin) (Sigma Aldrich, Saint-Louis, USA) pH 7.9, using a final volume of 15 μL at 37 °C for 2 h. Following this incubation, the material was loaded onto a 12% denaturing polyacrylamide gel electrophoresis (dPAGE). Two negative controls were used: a bacterial culture with empty vector (blank) and with *Bacillus subtilis* glycine oxidase (GO), expressed and purified under the same conditions as the CPR putative beta-lactamase-encoding genes. The Turbo DNase enzyme was also used as a positive control enzyme at a concentration of 1 U.

To evaluate ribonuclease activity, the RNA of each transformed *E. coli* was used as substrate and purified using RNeasy columns (Invitrogen, Carlsbad, CA, USA). The ribonuclease activities were performed by incubating RNA (1 µg) with 15 µg of purified putative beta-lactamase enzyme alone or in the presence of β-lactamase inhibitors (10 μg/mL sulbactam or 10 mM EDTA), in CutSmart Buffer, using a final volume of 15 µL at 37 °C for 2 h. After incubation, RNA-hydrolyzing activity was visualized on the RNA 6000 Pico LabChip (Agilent 2100 Bioanalyzer). The blank and the *B. subtilis* GO were also used as a negative control enzyme.

## 5. Conclusions

In conclusion, the CPR beta-lactamases could not be specifically evolved for beta-lactam resistance purposes. These enzymes are pleiotropic proteins with multifunctional hydrolase activities. Various substrates could be degraded by these beta-lactamase enzymes for possible further use of the metabolites as nutrient or carbon sources, as reported in bacteria [54,55,56,57].

## Figures and Tables

**Table 1:** Data for the CPR putative class A and class B beta-lactamase sequences tested in vitro.

Sequence Name	RAST Server Annotation	Repetition Number	E-Value	Length (a.a)	Confidence (%)	3D Structure Information
A-1	Hypothetical protein	16	6.02 × 10^4^	287	100	beta-lactamase class A like protein
A-2	Hypothetical protein	16	1.02 × 10^9^	303	100	beta-lactamase class A like protein
B-1	MBL-fold metallo-hydrolase superfamily	7	1.00 × 10^7^	263	100	Metal-dependent hydrolases of the beta-lactamase superfamily II
B-2	Hypothetical protein	13	3.38 × 10^4^	251	100	Metallo-beta-lactamase, human metallo-beta-lactamase containing protein 1
B-3	Hypothetical protein	4	3.83 × 10^4^	722	100	Hydrolase, Ribonuclease j 1; unusual, dual endo- and exonuclease activity in the degradosome2

## Data Availability

Not applicable.

## Supplementary Materials

The following supporting information can be downloaded at: https://www.mdpi.com/article/10.3390/ijms23105446/s1.
